# Dendritic-Tumor Fusion Cell-Based Cancer Vaccines

**DOI:** 10.3390/ijms17060828

**Published:** 2016-05-26

**Authors:** Shigeo Koido

**Affiliations:** Division of Gastroenterology and Hepatology, Department of Internal Medicine, The Jikei University School of Medicine, Kashiwa Hospital, 277-8567 Chiba, Japan; shigeo_koido@jikei.ac.jp; Tel.: +81-4-7164-1111

**Keywords:** dendritic cell, whole tumor cell, cell fusions, cancer vaccines, cytotoxic T lymphocyte

## Abstract

Dendritic cells (DCs) are potent antigen-presenting cells (APCs) that play a critical role in the induction of antitumor immunity. Therefore, various strategies have been developed to deliver tumor-associated antigens (TAAs) to DCs as cancer vaccines. The fusion of DCs and whole tumor cells to generate DC-tumor fusion cells (DC-tumor FCs) is an alternative strategy to treat cancer patients. The cell fusion method allows DCs to be exposed to the broad array of TAAs originally expressed by whole tumor cells. DCs then process TAAs endogenously and present them through major histocompatibility complex (MHC) class I and II pathways in the context of costimulatory molecules, resulting in simultaneous activation of both CD4^+^ and CD8^+^ T cells. DC-tumor FCs require optimized enhanced immunogenicity of both DCs and whole tumor cells. In this context, an effective fusion strategy also needs to produce immunogenic DC-tumor FCs. We discuss the potential ability of DC-tumor FCs and the recent progress in improving clinical outcomes by DC-tumor FC-based cancer vaccines.

## 1. Introduction

### 1.1. Dendritic Cell (DC)-Based Cancer Vaccines

T cell activation requires the processing of tumor-associated antigens (TAAs) into antigenic peptides, which are presented by antigen-presenting cells (APCs). Dendritic cells (DCs) are powerful APCs capable of inducing antitumor immune responses by linking the innate and adaptive immune systems [1,2]. Effective antigen processing and presentation by DCs are essential for the induction of antitumor immunity. There are three different pathways for antigen presentation by DCs. Exogenous antigens can be captured and processed into antigenic peptides by immature DCs, resulting in the cross-presentation of peptides on major histocompatibility complex (MHC) class I molecules [3,4]. Exogenous antigenic peptides can also be presented on MHC class II molecules on DCs [5]. In contrast, DCs can process endogenously synthesized antigens into antigenic peptides, which are presented on MHC class I molecules. DCs migrate to the draining lymph node where they present an antigen to CD4^+^ and CD8^+^ T cells, resulting in the induction of antigen-specific helper and cytotoxic T lymphocytes (CTLs), respectively. Moreover, interactions of DCs with innate and innate-like immune cells, such as natural killer (NK), invariant natural killer T (iNKT), and γδ T cells, generate crosstalk between DCs, resulting in efficient CTL induction [6,7,8]. Therefore, promising strategies to induce TAA-specific T cells have been developed using DCs [4]. In our laboratory, monocyte-derived immature DCs are generated by a single leukapheresis after culture in the presence of granulocyte macrophage colony-stimulating factor (GM-CSF) and interleukin (IL)-4, followed by activation with penicillin-killed and lyophilized preparations of a low-virulence strain (Su) of *Streptococcus pyogenes* (OK-432) and prostaglandin E2 (PGE2) [9]. A large number of mature DCs can then be cryopreserved in aliquots. However, the maturation of DCs with OK-432, PGE2, zoledronic acid, and tumor necrosis factor-α (TNF-α) may have more potential [10]. Thus far, mature DCs pulsed with specific TAA-derived peptides have been intensively investigated because they are simple and economical [11,12]. However, the major disadvantages of peptide-loading DC vaccines are related to the following factors: (1) the limited number of available immunogenic peptides specific for tumors [13]; (2) monoclonal peptide-specific CD8^+^ CTLs may not be sufficiently effective to treat cancer patients [14]; and (3) MHC class I molecules and certain TAAs are significantly downregulated in tumors during tumor progression [14]. Therefore, to induce polyclonal antigen-specific CTLs, DCs have also been loaded with TAAs in the form of tumor lysates [11], killed tumor cells [15], mRNA [16], and cDNA [17]. Moreover, an alternative strategy is the use of hybrid cells generated by the fusion of DCs and whole tumor cells (DC-tumor FCs), as first described by Gong *et al.* [18].

### 1.2. Fusions of Autologous DCs and Autologous Whole Tumor Cells

**Figure 1:**
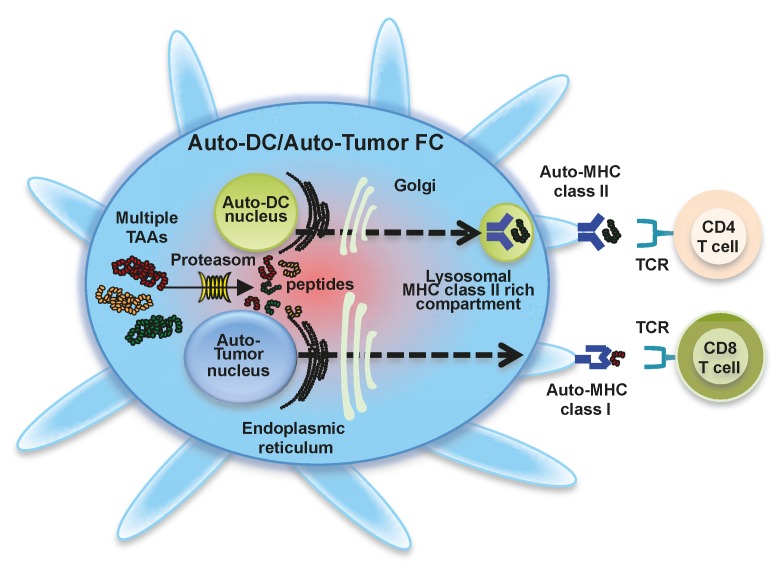
Fusion cells generated with dendritic cells and whole tumor cells. Fusions of dendritic cells (DCs) and whole tumor cells (DC-tumor FCs) display a characteristic phenotype comprised of major histocompatibility complex (MHC) class I molecules, MHC class II molecules, co-stimulatory molecules (CD80 and CD86), and multiple tumor-associated antigens. Multiple tumor-associated antigens from whole tumor cells are degraded by proteasome in DC-tumor FCs (solid line arrow), and antigenic peptides are loaded onto MHC class I molecules in the endoplasmic reticulum. The peptide-MHC class I complexes are then expressed on the DC-tumor FC surface (dash line arrow) and stimulate polyclonal antigen-specific CD8^+^ T cells. DC-tumor FCs can also synthesize MHC class II-restricted antigenic peptides in the endoplasmic reticulum. These are transported to the cytoplasm, where peptide-MHC class II complexes are generated (dash line arrow). Peptide-MHC class II complexes are also expressed on the DC-tumor FC surface and activate polyclonal antigen-specific CD4^+^ T cells.

The fusion of DCs and whole tumor cells by chemical, physical, or biological means creates heterokaryons, which include DC-derived MHC class I, MHC class II, and costimulatory molecules as well as whole tumor-derived large repertories of TAAs [19,20,21,22] (Figure 1). We have used polyethylene glycol (PEG) to generate DC-tumor FCs [18]. PEG-generated DC-tumor FCs display tight contact between the DC and tumor cell, thus, efficiently integrating these two cell types [20,23]. In general, mature DCs and whole tumor cells are mixed at a 5–10:1 ratio in pre-warmed serum-free RPMI 1640 medium. The mixed cell pellet is gently resuspended with pre-warmed PEG solution for 3–5 min at room temperature followed by dilution with pre-warmed serum-free RPMI medium. The cell pellet obtained by gentle centrifugation at room temperature is washed and cultured in the presence of GM-CSF, IL-4, and OK-432. On day five or six of culture, the loosely-adherent cells are collected by gently pipetting up and down several times. During culture, the DCs and whole tumor cells are integrated into a single entity [19,23,24]. Whole tumor-whole tumor fusion cells, as well as unfused whole tumor cells, grow firmly attached to the culture plates, whereas DC-tumor FCs, unfused DCs, and DC-DC FCs adhere loosely to the culture plates. Short-term culture of PEG-treated cell preparations can promote DC-tumor fusion efficiency [23]. Although fusion efficiency is low immediately after the fusion process, one week of culture provides DC-tumor FCs sufficient time to integrate and display antigen in the context of MHC molecules [23]. However, prolonged culture should be avoided because unfused tumor cells can overgrow. Fusion efficiency also depends on cell conditions due to the sensitivity of cells to PEG treatment. PEG treatment is most suitable for fusions of living cells [20]. Moreover, DCs can also capture apoptotic whole tumor cells during culture. Therefore, special methods are not necessary to enrich DC-tumor FC preparations [20]. In clinical trials, DC-tumor FC preparations have been irradiated to prevent proliferation of unfused tumor cells. Thus, irradiated DC-tumor FC preparations are incapable of spreading in cancer patients [22].

In DC-tumor FCs, the cytoplasm of both DCs and whole tumor cells is integrated without nuclear fusion, as demonstrated by immunoelectron microscopy (Figure 1) [19,23]. These morphological features allow the retention of the functions of both original cell types, including co-expression of tumor-derived whole TAAs (both known and unidentified) and DC-derived MHC class I and II molecules [20,23]. In general, DC-tumor FCs process multiple antigenic peptides from whole tumor cells and load them onto MHC class I molecules in the endoplasmic reticulum. The antigenic peptide-MHC class I complexes are expressed on the DC-tumor FC surface and presented to CD8^+^ T cells. The endogenous pathway of direct antigen processing and presentation in DC-tumor FCs is preserved. DC-tumor FCs can also synthesize MHC class II-restricted antigenic peptides from whole tumor cells in the endoplasmic reticulum. DC-derived MHC class II molecules and tumor-derived antigenic peptides travel by separate routes and converge to form MHC class II-peptide complexes in DC-tumor FCs, where MHC class II-antigenic peptide complexes are expressed on the DC-tumor FC surface and presented to CD4^+^ T cells. Therefore, polyclonal antigen-specific CD4^+^ and CD8^+^ T cells are directly induced by DC-tumor FCs in the draining lymph node [20,25,26,27,28,29,30,31]. Moreover, antigens derived from DC-tumor FCs may also be cross-presented by host DCs, resulting in the induction of antitumor immunity [25]. Importantly, the effector and memory CD4^+^ T cells induced by DC-tumor FCs are crucial for the maintenance of long-term antitumor immunity [31]. A subset of primed MUC1-specific CD4^+^ T cells induced by DC/MUC1-positive tumor FCs possesses cytotoxicity against MHC class I- and MUC1-positive tumor cells [31,32]. Moreover, adoptive transfer of primed CD4^+^ T cells prevents lung metastasis in Rag2−/−mice that lack NKT, T, and B cells due to an impaired T cell receptor (TCR) rearrangement [31]. Therefore, CD4^+^ T cell activation by DC-tumor FCs through MHC class II interactions plays an essential role in the induction of effective antitumor immunity.

In summary, the DC-tumor fusion approach offers the following advantages for inducing antitumor immune responses (Table 1): (1) DC-tumor FCs present whole tumor-derived antigenic peptides, which avoids the need to identify antigenic peptides for individual patients; (2) a broad array of known and unidentified TAAs can be simultaneously presented on the surface of DC-tumor FCs, which increases the frequency of polyclonal antigen-specific CD4^+^ and CD8^+^ T cells, resulting in long-term efficient antitumor immunity; (3) numerous TAAs are presented in the context of co-stimulatory molecules, which prevents tolerance induction, resulting in efficient antitumor immune response; and (4) DC-tumor FCs migrate into draining lymph nodes and form clusters with CD4^+^ and CD8^+^ T cells in the T cell area of lymph nodes, such that DC-tumor FCs do not have to take up exogenous TAAs in order to activate CD4^+^ and CD8^+^ T cells.

### 1.3. Fusion of Autologous DCs and Allogeneic Whole Tumor Cells

**Figure 2:**
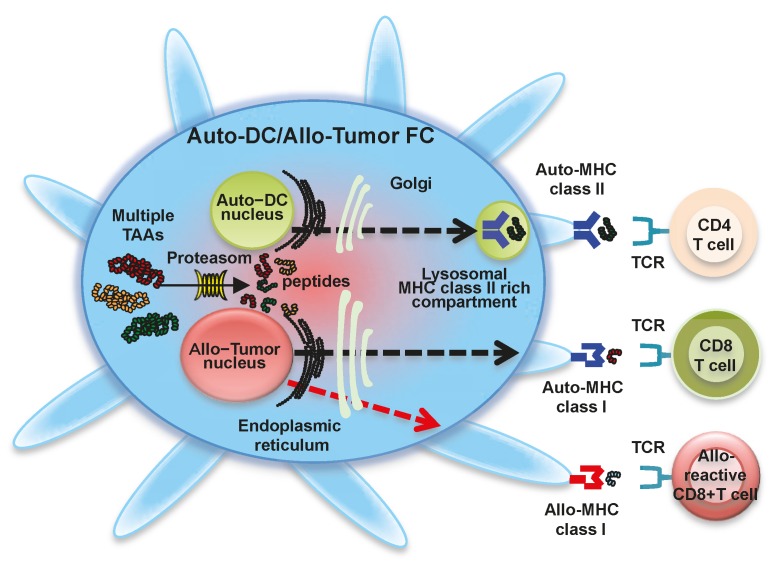
Fusion cells generated with autologous dendritic cells and allogeneic tumor cells. Fusions of autologous dendritic cells (Auto-DCs) and allogeneic tumor cells (Auto-DC/Allo-Tumor FC) display a characteristic phenotype comprised of major histocompatibility complex (MHC) class I molecules from autologous DCs and allogeneic tumor cells, MHC class II molecules from autologous DCs, co-stimulatory molecules (CD80 and CD86), and multiple tumor-associated antigens. Multiple tumor-associated antigens from allogeneic tumor cells are degraded by proteasome in Auto-DC/Allo-Tumor FC (solid line arrow), and antigenic peptides are loaded onto MHC class I molecules from autologous DC (black-colored dash line arrow) and allogeneic tumor cell (red-colored dash line arrow) in the endoplasmic reticulum. The peptide-MHC class I complexes are then expressed on the Auto-DC/Allo-Tumor FC surface and stimulate polyclonal antigen-specific CD8^+^ T cells and alloreactive CD8^+^ T cells. Auto-DC/Allo-Tumor FC can also synthesize MHC class II-restricted antigenic peptides in the endoplasmic reticulum. These are transported to the cytoplasm, where peptide-autologous MHC class II complexes are generated (black-colored dash line arrow). Peptide-autologous MHC class II complexes are also expressed on the Auto-DC/Allo-Tumor FC surface and activate polyclonal antigen-specific CD4^+^ T cells. Moreover, antigens derived from Auto-DC/Allo-Tumor FC are also be cross-presented by host DCs, resulting in the induction of polyclonal antigen-specific CD4^+^ and CD8^+^ T cells.

The main disadvantage of the DC-tumor fusion approach is the limited availability of viable autologous tumor cells as a fusion partner due to the long culture time of primary tumor cells, as well as potential bacterial and fungal contamination, especially in gastrointestinal tumors [33]. Thus, DC-tumor FC-based vaccines may need to shift from autologous tumor cells to easily applicable tumor cells. To circumvent this disadvantage, allogeneic tumor cell lines have been used in place of autologous tumor cells to induce autologous tumor-specific antitumor immune responses (Figure 2) [33,34,35]. The scientific basis for this approach is that almost all allogeneic tumor cell lines share certain TAAs with autologous tumor cells. Antigenically well-defined allogeneic tumor cell lines sharing TAAs with autologous tumor cells can proliferate well *in vitro* under good manufacturing practice (GMP) standards, thus generating sufficient numbers of available tumor cells for a fusion partner. Moreover, allogeneic tumor cell lines are highly standardized in large-scale production vaccines [36]. For the DC-tumor fusion strategy, allogeneic tumor cell lines can theoretically process and present multiple TAAs through MHC class I and II molecules from autologous DCs. Our previous study indicated that fusions of autologous DCs and allogeneic tumor cell lines (e.g., colorectal cancer cells, breast cancer cells, or pancreatic cancer cells) present multiple TAAs and induce antigen-specific CTL responses restricted by autologous HLA [33,34,35]. The phenomenon of cross-priming against shared TAAs allows the use of allogeneic tumor cell lines to present antigenic peptides to autologous DCs in fusions of autologous DCs and allogeneic tumor cells. Therefore, it is not necessary to match HLA types between cancer patients and allogeneic tumor cells used for generating fusions [33,34]. In addition, allogeneic MHC molecules from tumor cell lines may induce allogeneic responses, promoting effective antitumor immunity [37]. Thus far, clinical trials have not reported using DC-tumor FCs generated with allogeneic tumor cell lines.

### 1.4. Fusion of Allogeneic DCs and Autologous Whole Tumor Cells

**Figure 3:**
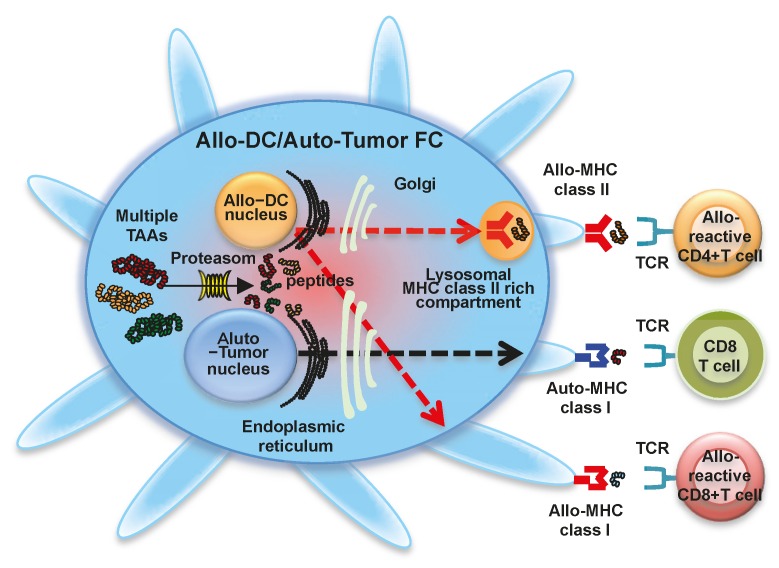
Fusion cells generated with allogeneic dendritic cells and autologous tumor cells. Fusions of allogeneic dendritic cells (Allo-DCs) and autologous tumor cells (Allo-DC/Auto-Tumor FC) display a characteristic phenotype comprised of MHC class I molecules from allogeneic DCs and autologous tumor cells, MHC class II molecules from allogeneic DCs, co-stimulatory molecules (CD80 and CD86), and multiple tumor-associated antigens. Multiple tumor-associated antigens from autologous tumor cells are degraded by proteasome in Allo-DC/Auto-Tumor FC (solid line arrow), and antigenic peptides are loaded onto MHC class I molecules from allogeneic DC (red-colored dash line arrow) and autologous tumor cell (black-colored dash line arrow) in the endoplasmic reticulum. The peptide-MHC class I complexes are then expressed on the Allo-DC/Auto-Tumor FC surface and stimulate polyclonal antigen-specific CD8^+^ T cells and alloreactive CD8^+^ T cells. Allo-DC/Auto-Tumor FC can also synthesize MHC class II-restricted antigenic peptides in the endoplasmic reticulum. These are transported to the cytoplasm, where peptide-allogeneic MHC class II complexes are generated (red-colored dash line arrow). Peptide-allogeneic MHC class II complexes are also expressed on the Allo-DC/Auto-Tumor FC surface and activate alloreactive CD4^+^ T cells. Moreover, antigens derived from Allo-DC/Auto-Tumor FC are also be cross-presented by host DCs, resulting in the induction of polyclonal antigen-specific CD4^+^ and CD8^+^ T cells.

Cancer patient-derived DCs are sometimes defective as APCs due to tumors, tumor microenvironments, and cancer therapies [38]. Therefore, the use of DCs from healthy donors as a source of allogeneic DCs to generate allogeneic DC-tumor FC vaccines has been investigated (Figure 3) [39]. Advantages of this strategy are that DCs generated from healthy donors have functional APCs and are readily available in sufficient quantities. Allogeneic DC-tumor FCs express DC-derived allogeneic HLA class II molecules for direct stimulation of alloreactive CD4^+^ T cells. This allogeneic response may contribute to enhanced and maintained antigen-specific CTL responses by cytokines from alloreactive CD4^+^ T cells [37]. We have generated allogeneic DC-tumor FCs in murine [40,41,42], preclinical [43], and clinical [44] studies, and we have shown equal or less potential of allogeneic DC-tumor FCs with autologous DC-tumor FCs. If an allogenically-healthy donor and cancer patient do not share any HLA molecules, the patient’s MHC-restricted presentation of antigenic peptides by allogeneic DC-tumor FCs is not possible. If the HLA typing is shared between the healthy donor and cancer patient, the antigen-specific CTL induction changes dramatically [37]. Where there is some sharing of MHC class I molecules, the alloreactive CD4^+^ T cell response provides potent T cell help for the generation of CD8+ CTL responses to tumor peptides presented by the shared HLA class I molecules. Antigen-specific CD4^+^ and CD8^+^ T cell responses against autologous tumor cells induced by allogeneic DC-tumor FCs are dependent on HLA type. Therefore, fusions of autologous DCs and autologous tumor cells may be more effective in targeting tumor cells because they can stimulate antigen-specific CD4^+^ T cells.

### 1.5. Fusion of Allogeneic DCs and Allogeneic Whole Tumor Cells

**Figure 4:**
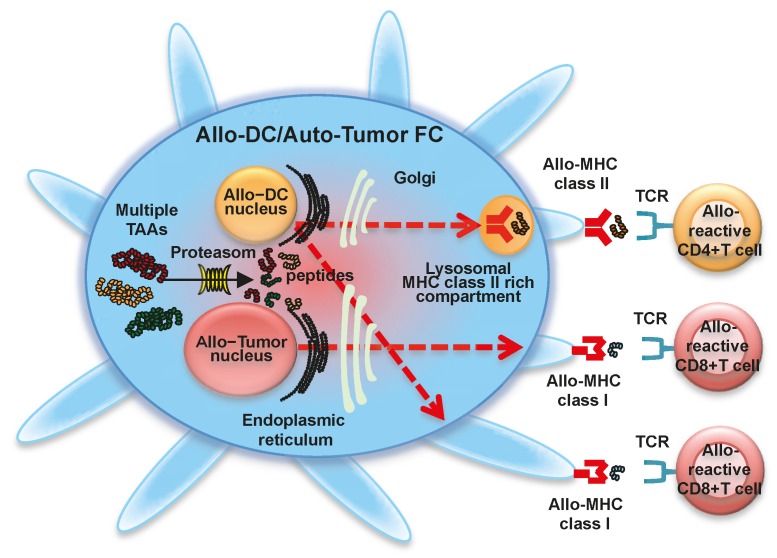
Fusion cells generated with allogeneic dendritic cells and allogeneic tumor cells. Fusions of allogeneic dendritic cells (Allo-DCs) and allogeneic tumor cells (Allo-DC/Allo-Tumor FC) display a characteristic phenotype comprised of MHC class I molecules from allogeneic DCs and allogeneic tumor cells, MHC class II molecules from allogeneic DCs, co-stimulatory molecules (CD80 and CD86), and multiple tumor-associated antigens. Multiple tumor-associated antigens from allogeneic tumor cells are degraded by proteasome in Allo-DC/Allo-Tumor FC (solid line arrow), and antigenic peptides are loaded onto MHC class I molecules from allogeneic DC (red-colored dash line arrow) and allogeneic tumor cell (red-colored dash line arrow) in the endoplasmic reticulum. The peptide-MHC class I complexes are then expressed on the Allo-DC/Allo-Tumor FC surface and stimulate alloreactive CD8^+^ T cells. Allo-DC/Allo-Tumor FC can also synthesize MHC class II-restricted antigenic peptides in the endoplasmic reticulum. These are transported to the cytoplasm, where peptide-allogeneic MHC class II complexes are generated (red-colored dash line arrow). Peptide-allogeneic MHC class II complexes are also expressed on the Allo-DC/Allo-Tumor FC surface and activate alloreactive CD4^+^ T cells. Moreover, antigens derived from Allo-DC/Allo-Tumor FC are also be cross-presented by host DCs, resulting in the induction of polyclonal antigen-specific CD4^+^ and CD8^+^ T cells.

Allogeneic DC lines and allogeneic tumor cell lines may be used instead of autologous cells. Cell lines are well characterized and can be well propagated *in vitro* under GMP standards. Therefore, unlimited amounts of allogeneic DC-allogeneic tumor FCs can be readily available (Figure 4). DC-tumor FC vaccines with fully allogeneic components have been demonstrated to induce clinical responses [45]. Moreover, we have shown that fusion cells generated with an allogeneic DC line and allogeneic tumor cell line can induce antigen-specific CTL responses *in vitro* [46]. These findings introduce the possibility of using defined allogeneic DCs and allogeneic tumor lines to induce antigen-specific CTLs for adoptive immunotherapy. Although DC-tumor FCs generated with fully syngeneic components may be most effective for antigen-specific CTL induction, semi-allogeneic and fully-allogeneic components may also have potential in the field of adoptive immunotherapy [47].

### 1.6. Fusion of Activated Autologous DCs and Autologous Whole Tumor Cells

Although DC-tumor FC vaccines are safe and have induced efficient antitumor immune responses in early clinical trials, a limited number of efficient clinical responses has been reported in glioblastoma, myeloma, melanoma, gastric cancer, breast cancer, or renal cell cancer patients [21,22,39,48]. To maximize the induction of antitumor immunity by vaccination with DC-tumor FCs, many types of adjuvants have accompanied DC-tumor FCs in animal studies. For example, adjuvants, including IL-2 [49], IL-12 [50,51,52], IL-18 [52,53], oligodeoxynucleotides containing a CpG motif (CpG ODN) [53,54], 1-MT (an indoleamine-pyrrole 2,3-dioxygenase inhibitor) [55], or polyriboinosinic polyribocytidylic acid [Poly(I:C)]/IL-10 siRNA, have been used [56]. Therefore, in clinical trials, DC-tumor FCs must be co-administered with adjuvants to induce efficient clinical responses in advanced cancer patients. Despite the small quantity of FCs, vaccination using DC-tumor FCs with IL-12 has demonstrated better therapeutic responses compared with DC-tumor FCs alone [57,58]. Co-administration of IL-12 with DC-tumor FCs may promote antitumor T helper type 1 (Th1) polarization, resulting in the activation of NK cells and antigen-specific CTLs [59].

Modification of DC-tumor FCs is also required to treat advanced cancer patients. Interestingly, both DCs and whole tumor cells can be independently immunomodulated while maintaining their individual characteristics even after fusion. This characteristic of DC-tumor FCs is advantageous compared to the strategy of whole tumor cell lysate loading of DCs. Recognition of microbial stimuli by Toll-like receptors (TLRs) expressed on DCs is effective for generating immunogenic DCs, leading to the production of Th1 cytokines, such as IL-12, and co-stimulatory molecules, such as CD80 and CD86, in DCs [60,61]. Full activation of DCs requires receptor signaling by combined TLR agonists [62]. The combination of TLR2 and TLR4 agonists has been shown to promote the immunogenicity of fusions, as demonstrated by the following results: (1) increased fusion efficiency; (2) upregulation of MHC class II molecules, heat shock proteins (HSPs), and CD86 expression in DC-tumor FCs; (3) increased IL-12 production from DC-tumor FCs; (4) activation of antigen-specific polyclonal CD4^+^ and CD8^+^ T cells producing high levels of interferon-γ (IFN-γ); and (5) induction of augmented antitumor immunity [39,63,64]. Therefore, the combination of TLR2 and TLR4 agonists for DC activation is advantageous and has potential application in immunogenic fusions for cancer vaccines.

### 1.7. Fusion of Autologous DCs and Immunogenic Autologous Whole Tumor Cells

Whole tumor cells are also required for the generation of immunogenic DC-tumor FCs. Indeed, many kinds of tumor cells produce immunosuppressive molecules, such as transforming growth factor β (TGF-β), IL-10, and vascular endothelial growth factor (VEGF). In particular, TGF-β1 is one of the major cytokines that impair DC function [65] and generate regulatory T cells (Tregs) [66], which play an important role in the inactivation of antitumor immunity [67]. TGF-β1 produced from whole tumor cells decreases antitumor immunity by DC-tumor FCs even when TLR2 and TLR4 are co-administered [64]. Therefore, several strategies have been developed to improve poor immunogenicity in tumor cells. In one fusion strategy, TGF-β production from whole tumor cells is blocked by a soluble TGF-β receptor expressing DC-tumor FCs, resulting in reduced Treg generation and augmented antitumor immunity [68]. As compared to conventional DC-tumor FCs, we have previously demonstrated that fusions generated with DCs and heat- [63] or ethanol-treated [69] tumor cells upregulate multiple HSPs, MHC class I molecules, MHC class II molecules, TAAs, CD80, CD86, and IL-12. Therefore, the fusion of DCs and immunogenic whole tumor cells activates antigen-specific CD4^+^ and CD8^+^ T cells that produce high levels of IFN-γ. Therefore, immunogenic whole tumor cells induced by heat or ethanol treatment enhance the immunogenicity of DC-tumor FCs as cancer vaccines. Interestingly, TGF-β1 production from tumor cells is also easily blocked by exposure to ethanol without downregulating MHC class I molecules and TAAs [69]. Moreover, inducing immunogenic tumor cell death by ethanol treatment ectopically induces calreticulin and high-mobility group box 1 (HMGB1), which activate DCs [70,71]. Immunogenic tumor cells might be used as whole tumor cell-based cancer vaccines. In clinical trials, allogeneic tumor cell lines have been transduced with GM-CSF to generate immunogenic whole tumor cells to be used for cancer vaccines (GVAX), leading to augmented antitumor immunity in early clinical trials [72]. One disadvantage of using heat- or ethanol-treated whole allogeneic tumor cells is the necessary dose response test to evaluate the optimal conditions for generating immunogenic whole tumor cells. The fusion strategy generated with activated DCs by TLRs and immunogenic whole tumor cells synergistically yields efficient TAA processing and presentation by DC-tumor FCs, with upregulated expression of MHC class I molecules, MHC class II molecules, co-stimulatory molecules, TAAs, and IL-12 [63,73,74]. However, it remains unclear which specific agents, including cytotoxic chemotherapeutic agents, or irradiation processes are best suited to generate immunogenic tumor cells. Thus, optimal strategies to maximize the clinical outcomes of DC-tumor FCs-based cancer vaccines must be identified.

### 1.8. Fusion of DCs and Cancer Stem Cells

It is well accepted that cancer stem cells (CSCs) are resistant to standard therapies, such as chemotherapy and irradiation [75,76]. Therefore, small populations of chemoresistant CSCs are responsible for lethal events, including tumor relapse and growth, due to therapeutic failure [75,76]. Importantly, chemoresistant CSCs preferentially express stem cell markers, including OCT3/4, ABCG2, nestin, SOX2, Bmi-1, Notch-1, CD44, CD133, and CD177 [75,77]. Moreover, CSCs overexpress survivin, MUC1, hTERT, HER2, CERP55, COA-1, and WT1 [77,78]. Thus, CSCs remain attractive targets for cancer vaccines, and the success of cancer vaccines may at least partly depend on the efficient induction of anti-CSC immunity. We have reported that fusions of DCs and CD44^+^ tumor cells with CSC characteristics (e.g., spheroid formation in culture, self-renewal, and an ability to be engrafted in immunocompromised mice) can endogenously process and present multiple CSC-specific antigenic peptides on MHC class I and II molecules, resulting in the induction of efficient CSC-specific CTL responses [76]. Moreover, another study demonstrated that fusions of DCs and CD133^+^ tumor cells could induce antitumor immunity similar to that of DC and CD133-tumor cell fusions [79]. Fusion cells generated with DCs and CSCs (DC-CSC-FCs) can process and present whole CSC antigens, thus avoiding the need for identification of CSC-specific antigens. Therefore, DC-CSC-FC vaccines may be an alternative approach to induce efficient anti-CSC immunity. Interestingly, MUC1 expression is upregulated in chemoresistant CSCs that are efficiently lysed by MUC1-specific CTLs in mice [76,80]. Therefore, DC-CSC-FCs should be combined with conventional chemotherapy.

### 1.9. Chaperone-Peptide Complexes from DC-Tumor Fusion Cells (FCs)

Cancer vaccines based on HSP-peptide complexes are promising because molecular chaperones, such as HSP70 and HSP90, have been shown to form complexes with a wide panel of peptides, including antigenic peptides [81,82]. HSP-peptide complexes can be taken up by DCs through specific receptors, facilitating DC activation and promoting antigen processing and presentation by MHC class I and II molecules on the DC surface [83]. We and other groups have attempted to prepare cancer vaccines based on HSP-peptide complexes derived from DC-tumor FCs [84,85,86,87]. We have demonstrated that HSP70-peptide complexes (HSP70.PC) extracted from DC-tumor FCs (HSP70.PC-FCs) have a superior ability to activate DCs and induce antigen-specific CTLs compared to HSP70.PC from whole tumor cells (HSP70.PC-T) in mice [84] and in preclinical studies [85]. Therefore, the DC-tumor FC strategy is useful in the preparation of HSP70.PC-based cancer vaccines in patients. This alternative approach relies on the efficient antigen processing and presentation machinery of DC-tumor FCs [85].

### 1.10. DC-Tumor FC Combination Therapy

As standard cancer therapies, such as chemotherapy, radiotherapy, and targeted therapies, interfere with DNA synthesis of tumor cells and peripheral lymphocytes, the therapy may blunt antitumor immune responses. However, increasing evidence suggests that some chemotherapeutic agents have the potential to augment cancer vaccines. Chemotherapy agents or irradiation can promote intrinsic immunogenicity of whole tumor cells in multiple ways. For example, sublethal therapies undergo immunogenic modulation and demonstrate upregulation of various danger signals, such as HMGB1, HSP70/90, adenosine triphosphate (ATP), and calreticulin (CRT) [70,88,89,90]. CRT is a Ca^2+^-binding chaperone that is found in different apoptotic stages and leads to increased cross-priming of antitumor T cell immunity without Treg induction [70,88,89,91,92,93]. Moreover, HMGB1 and HSP70/90 from immunogenic tumor cells interact with TLR4 on DCs, which activates the antigen processing and presentation machinery in DCs [71,94,95]. Some therapies also upregulate TAAs, such as WT1, CEA, MUC1, and Her2, as well as MHC class I molecules [96,97,98,99]. Finally, a positive interaction of DC-tumor FC-based cancer vaccines with chemotherapy or radiotherapy may generate a new era for cancer immunotherapy. Moreover, chemotherapy- or radiotherapy-induced immunogenic tumor modulation is a better candidate as a fusion partner than conventional DC-tumor FCs.

### 1.11. Future Cancer Regimens Using DC-Tumor FCs

Effective and selective targeted therapies with little toxicity are urgently needed for patients with advanced cancer. Treatment of cancer patients with DC-tumor FCs alone has limitations due to the immunosuppressive mechanism. DC-tumor FCs can induce antigen-specific CTLs and Tregs [100]. It is well known that some chemotherapeutic agents, such as cyclophosphamide and gemcitabine, can activate antitumor immunity by depleting Tregs and myeloid-derived suppresser cells (MDSCs) [101,102], resulting in efficient clinical outcomes. Recent reports have also indicated that vaccination induces programmed death 1 (PD1) expression in activated CTLs [103]. The PD1 ligand, PD-L1, in tumor cells is upregulated by IFN-γ produced by activated CTLs. As a result, PD1-PD-L1 signaling is associated with impaired CTL function. A preclinical study has shown that an anti-PD1 antibody decreases Tregs and enhances CTL activity [104]. Moreover, inactivation of CD4^+^CD25^+^Foxp3^+^ Tregs by an anti-CD25 antibody following DC-tumor FC vaccination significantly improves antitumor immunity in a murine model [105]. Therefore, a therapeutic regimen combining DC-tumor FCs, chemotherapy, Treg depletion, and antibody blockade of PD1-PD-L1 signaling may have potential in advanced cancer patients [21,106]. Such multiple immune checkpoint blockades to facilitate CTL induction without T cell anergy in the presence of DC-tumor FC-augmented CTLs may be the most efficient treatment strategy. It is important to understand which combinations of immunoinhibitory molecules and which patients are suitable for the therapies [107].

## 2. Conclusions

We developed DC-tumor FCs strategies to induce efficient antitumor immune responses mediated by antigen-specific CD4^+^ and CD8^+^ T cells and to break T cell tolerance to TAAs. Although DC-tumor FCs strategy has numerous advantages, the clinical responses to DC-tumor FCs are not as vigorous as in the animal tumor models. Therefore, DC-tumor FCs require optimized enhanced immunogenicity of both DCs and whole tumor cells to maximize their immunogenicity. Moreover, the combination of DC-tumor FCs with standard therapies such chemotherapy and irradiation may provide robust clinical benefits. Specially, an immune checkpoint blockade combined with DC-tumor FCs may be effective in treating patients with advanced cancer. It is also essential to develop strategies targeting immunosuppressive microenvironment of tumors.

## Figures and Tables

**Table 1:** Advantages and disadvantages of DC-tumor FCs.

Advantages	DC-tumor FCs present whole tumor-derived antigenic peptides, which avoids the need to identify antigenic peptides for individual patients.
	A broad array of known and unidentified tumor-associated antigens are simultaneously presented on the surface of DC-tumor FCs.
	Endogenously-synthesized tumor-associated antigens in DC-tumor FCs are better access to MHC class I and II molecules.
	Increased the frequency of polyclonal antigen-specific CD4^+^ and CD8^+^ T cells can be induced by DC-tumor FCs.
	DC-tumor FCs can induce long-term efficient antitumor immunity.
	Numerous tumor-associated antigens are presented in the context of co-stimulatory molecules in DC-tumor FCs.
	DC-tumor FCs prevent tolerance induction.
	Autologous DC-autologous tumor FCs do not have to take up exogenous TAAs in order to activate CD4^+^ and CD8^+^ T cells.
	Modifications of DCs and tumor cells are independently possible while their characters present after the fusion.
	Allogeneic DC and allogeneic tumor cells can be used instead of autologous cells in generation of DC-tumor FCs.
	DC-tumor FC-based cancer vaccines can be combined with standard therapies.
Disadvantages	The limited availability of viable autologous tumor cells as a fusion partner.
	Induction of antigen-specific CD4^+^ and CD8^+^ T cell responses by allogeneic DC-tumor FCs are at least partly associated with sharing of MHC class I.
	Fusion efficiency depends on cell conditions due to the sensitivity of cells to PEG treatment.

DC-tumor FCs: fusions of dendritic cells and whole tumor cells; MHC: Major histocompatibility complex; PEG: Polyethylene glycol.

## Abbreviations

The following abbreviations are used in this manuscript:

ATP: Adenosine triphosphate
APCs: Antigen presenting cells
CRT: Calreticulin
CSCs: Cancer stem cells
CTLs: Cytotoxic T lymphocytes
DCs: Dendritic cells
DC-tumor FCs: Fusions of DCs and whole tumor cells
GMP: Good manufacturing practice
GMP: Granulocyte macrophage colony-stimulating factor
HSPs: Heat shock proteins
HSP70.PC: HSP70-peptide complexes
HSP70.PC-FCs: HSP70.PC from DC-tumor FCs
HSP70.PC-T: HSP70.PC from whole tumor cells
IFN-γ: Interferon-γ
IL: Interleukin
iNKT: Invariant natural killer T
MHC: Major histocompatibility complex
MDSCs: Myeloid derived suppresser cells
NK: Natural killer
Poly(I:C): Polyriboinosinic polyribocytidylic acid
PEG: Polyethylene glycol
PD1: Programmed death 1
PD-L1: Programmed death ligand 1
PGE2: Prostaglandin E2
Tregs: Regulatory T cells
Th1: T helper type 1
TLR: Toll-like receptor
TGF-β: Transforming growth factor β
TAAs: Tumor-associated antigens
TNF-α: Tumor necrosis factor-α
VEGF: Vascular endothelial growth factor
